# A Shared Focus: Comparing the Australian, Canadian, United Kingdom and United States Pharmacy Learning Outcome Frameworks and the Global Competency Framework

**DOI:** 10.3390/pharmacy4030026

**Published:** 2016-09-10

**Authors:** Ieva Stupans, Jeffrey Atkinson, Arijana Meštrović, Rose Nash, Michael J. Rouse

**Affiliations:** 1 School of Health and Biomedical Sciences, RMIT University, P.O. Box 71, Bundoora, Victoria 3083, Australia; 2 Pharmacolor Consultants Nancy, 12 rue de Versigny, Villers 54600, France; Jeffrey.Atkinson@univ-lorraine.fr; 3 Pharma Expert Consultancy and Education, Dunjevac 2, 10000 Zagreb, Croatia; arijana.mestrovic@pharmaexpert.hr; 4 Faculty of Health, University of Tasmania, Hobart, Tasmania 7001, Australia; rose.mcshane@utas.edu.au; 5 Accreditation Council for Pharmacy Education, 135 South LaSalle Street, Suite 4100, Chicago, IL 60603-4810, USA; mrouse@acpe-accredit.org

**Keywords:** competencies, Global Competency Framework, GbCF, learning outcomes, mapping

## Abstract

This paper presents an analysis of the end of degree expectations, expressed as learning outcomes, for pharmacy graduates from Australia, Canada, United Kingdom and United States. The authors compare the end of degree expectations, through mapping these requirements to the International Pharmaceutical Federation (FIP) Global Competency Framework (GbCF). The anticipated end of degree expectations are similar but also reveal some individual characteristics. Irrespective of degree title, achievement of learning outcomes specified in any one of the four jurisdictions should enable students to become pharmacists who are patient-orientated medicines experts. The mapping provides impetus for cross-border institutional networking to generate a dependable set of assessment tools across national borders developing a common metric for outcome assessment irrespective of different program delivery.

## 1. Introduction

The needs-based pharmacy education model [1] proposes that pharmacy education programs are designed such that pharmacy graduates are able to deliver pharmacy services that meet the needs of national populations. Australia, Canada, the United Kingdom and the United States are all high income developed countries with very similar life expectancies (83, 82, 81 and 79 respectively, 2012 data [2]) and with similar national health-related needs. For these four countries, six of the ten top causes of death are identical (2012 data [2]) and in these four countries pharmacists are broadening their roles as health care providers, for example as pharmacist vaccinators [3,4,5,6]. Given the similar service needs how do the expected graduate learning outcomes compare?

For the purposes of this paper the term outcomes will be used for end of degree requirements. Learning outcomes are “a statement of what a learner is expected to know, understand and be able to do at the end of a period of learning” [7] emphasising the application of the ability, capacity or skill to accomplish a task. Outcomes provide a way to communicate external reference points at the national and international levels both within and outside the profession. Outcomes help faculty and other stakeholders such as employers to have a common understanding about the specific skills and knowledge that graduates should have mastered as a result of their learning experiences and promote a shared and common understanding of the expectations associated with typical qualifications. Learning outcomes are threshold, indicating outcomes for pass grade students—they provide a benchmark, they do indicate how successful a student was in achieving the outcome [8].

Curriculum design in this case is based around students’ achievement of learning outcomes [9] which can be assessed and assured at the end of degree. Competencies for pharmacists in practice and educational outcomes for new graduates are strongly correlated; however, the actual relationship between students’ achievement of threshold outcomes and successful complex professional performance in the workplace is unclear. Competencies may be further developed through professional practice.

The four jurisdictions examined in this paper have different requirements for registration as a practicing pharmacist. This in turn affects the requirements for enabling degrees. In Australia, completion of a one year internship is required irrespective of the qualification degree level (Bachelor or Masters degree); in Canada there is a mix of both Bachelor and Doctor of Pharmacy Programs [10] with differing requirements for internship. In the United Kingdom, a four year Masters degree is required in addition to a 52 week pre-registration training—although the option of a five year Masters degree with two intercalated periods of pre-registration training has also been approved [11]. Post degree practice requirements reflect the curriculum in each jurisdiction. For example, in the United States a significant portion of the Doctor of Pharmacy curriculum is now practice based and, accordingly, for most states, no additional structured postgraduate practice-based experience is required prior to registration (licensure).

The paradigm shift to students’ achievement of learning outcomes has both academic and political implications. First, it can be argued that good curricular design relies on alignment. In an aligned curriculum, innovation in curriculum design is not constrained by traditional arguments around credit points of content; the arrangement of programs is determined by what students should be able to do at the completion of the program. Every course in the curriculum needs to be mapped to a specific educational outcome(s). The mapping exercise should also highlight how the curricula structure and course sequencing facilitate the progressive development of each educational outcome.

Second, the political agenda calls for demonstrable quality and impact (“social accountability”) given significant financial investment in the higher education sector along with the quest for increased participation, diversity with respect to participation, recognition of prior learning and student mobility. In Australia, the Australian Qualifications Framework (AQF) [12] underpins national regulatory and quality assurance arrangements for education and training, characterising the knowledge and skills a graduate should have and be able to apply at each different qualification level. In Canada, a ministerial council statement provides a context for identifying how degree credentials compare in level and standard to those in other jurisdictions [13]. In the United Kingdom, the Quality Assurance Agency for Higher Education (QAA) is the independent body entrusted with monitoring standards and quality in higher education and provides a framework on degree requirements [14]. The arrangement in the United States is somewhat different in that the U.S. Department of Education establishes policy for, administers and coordinates most federal assistance to education, but the Constitution gives individual states authority over education; institutions of higher education are permitted to operate with considerable independence and autonomy. Accrediting agencies, of regional or national scope, develop evaluation criteria and undertake evaluations. For example, the Accreditation Council for Pharmacy Education (ACPE) is approved by the Department of Education for the accreditation of professional degree programs in pharmacy, and has specified educational outcomes for accreditation [15].

Thus, in Australia, Canada and the United Kingdom national guidelines regarding levels for all tertiary qualifications across all disciplines set a broad standards framework, which is interpreted at the individual profession level. In the United States, ACPE sets a profession based standards framework. However, across all of these jurisdictions teaching teams construct the curriculum, selecting content and designing and manage teaching, learning and assessment arrangements.

In Australia and Canada, responding to both academic and political imperatives, projects have been undertaken to develop frameworks outlining university expectations of pharmacy graduates [16,17,18]. In these jurisdictions, work to develop a set of expected educational outcomes for pharmacy programs has included initial conceptualisation followed by a process of validation through feedback from a number of stakeholders including discipline teachers, practitioners and students, and subsequent achievement of consensus.

In Australia, federal policy has directed that discipline communities take responsibility for implementing teaching and learning standards (working with professional bodies and other stakeholders where appropriate) [19] (p. 32). Federal funding was provided for the development of threshold learning outcomes that could be applied to students graduating from any Australian University program across a number of disciplinary groupings (e.g., Science, Education). As an extension of this initial work, a federally funded nationwide Pharmacy discipline network developed learning outcomes and exemplar standards for programs preparing pharmacy professionals [16]. Six of the eight learning outcomes are common with those of medicine, nursing and other health professionals whereas two of the eight are pharmacy specific, referring to the role of the current graduate regarding formulation, preparation, and supply of medications and therapeutic products and the application of pharmaceutical, medication and health knowledge and skills [16].

In Canada, the Association of Faculties of Pharmacy of Canada has produced a single set of educational outcomes for first professional degree programs in pharmacy [17,18] using format and terminology from the CanMeds [20] model. The CanMeds model is a competency based education framework focused on defining the key roles and competencies required by medical physicians; however, it has also been extended to other health disciplines, such as nursing, medical radiation and physiotherapy [21,22]. Pharmacy graduates are considered “Medication Therapy Experts”, able to integrate knowledge, skills and attitudes under the roles of Care Provider, Communicator, Collaborator, Manager, Advocate, Scholar, and Professional.

In the United Kingdom, outcomes are specified by the General Pharmaceutical Council [11] and are included in program accreditation requirements; however, it is important to note that standards are set with extensive consultation.

In the United States, a stakeholder/consensus-based process convened by the American Association of Colleges of Pharmacy has been used to develop the Centre for the Advancement of Pharmacy Education (CAPE) Educational Outcomes [23]. The fourth version (2013), has been incorporated into ACPE’s Pharmacy Degree Program Accreditation Standards. To guide their work, the CAPE 2013 panel used literature from pharmacy and other health professions and gained additional perspectives from other health professions and a patient care advocate. CAPE 2013 was intentionally expanded beyond knowledge and skills to include the affective domain.

A global accord on foundation level career education and training [24]—the Global Competency Framework (GbCF), which was developed by the Education Initiative (FIP*Ed*) of the International Pharmaceutical Federation (FIP), represents and thus provides, a global framework of consensus knowledge, skills and attributes at the practitioner foundation level. The development of this framework involved the analysis of eight different (from different countries) pharmacy practitioner development competency frameworks and synthesis of core elements which were further categorized into the domains of Pharmaceutical Public Health, Pharmaceutical Care, Organisation and Management, and Professional/Personal. The GbCF has been proposed as having applicability for the fostering of transnational collaboration and has been used as a mapping and assessment tool [25].

The purpose of this paper is to review the level of commonality and the differences between expectations of pharmacy graduates, articulated as outcomes from four different jurisdictions—Australia, Canada, the United Kingdom and the United States, aligning these to the GbCF—the global competency mapping tool. It examines at a multi jurisdiction level the alignment of pharmacy workforce preparatory education (learning outcomes) and global pharmacy workforce practice (competencies).

## 2. Method

The method utilised in this study was qualitative description [26]. A comparative document analysis [27] mapping learning outcomes from each jurisdiction to the GbCF was undertaken by one author and checked by others; revision of alignment was undertaken until consensus was reached, essentially through online communication, suggestions and feedback, with all manuscript authors. Briefly the process involved superficial, then thorough examination and interpretation of the documents. The GbCF domains were used as predefined categories for analysis. Each jurisdiction’s learning outcomes were compared methodically considering similarities and differences with respect to each of the GbCF domains, thus enabling Table 1 to be populated with the learning outcomes from the four jurisdictions mapped against the GbCF. The audit trail for this study incorporates documentation of the decisions made during the project. There were four rounds of editing of the alignment. This method was selected as a means of affording detailed aligned information across the four jurisdictions.

## 3. Results

Content analysis of high-level outcomes from Australia, Canada, the United Kingdom and United States with the GbCF is shown in Table 1. The noteworthy finding is the overall alignment of the same basic elements of public health, pharmaceutical care and personal attributes as described within the GbCF with the learning outcomes described in each jurisdiction’s framework.

Some differences across the four jurisdictions are evident. For example, dispensing receives little emphasis, compounding no emphasis in the Canadian, United Kingdom and United States outcomes as compared to the Australian outcomes. This may be a consequence of an individual country’s industrial regulations around the work of pharmacy technicians. Australian outcomes do not include organisation and management potentially reflecting the Australian system of an internship year post-graduation, pre-registration.

## 4. Discussion

Considering the similarity of health needs across Australia, Canada, the United Kingdom and the United States, it is not surprising that educational programs that aim to prepare a workforce that is able to fulfil these needs are similar; i.e., needs based education [28]. Elements such as communication with patients, personal behaviours, safe and effective practice and currency/renewing of practice and continuing professional education/development appear in all educational learning outcomes frameworks as well as the GbCF. 

The development of the GbCF involved the analysis of a number of competency frameworks and synthesis of core elements. Four of these eight practitioner development competency frameworks were from Australia, Canada, the United Kingdom and United States, thus similarity across jurisdictions and the GbCF is not surprising. However, it is important to note the recency of the learning outcomes documents that were examined in this paper [16,17,18] as opposed to the competency frameworks analysed in the GbCF.

Of concern given the rapidly changing global health environment, is that of the four jurisdictions and the GbCF only the United States outcomes specify innovation and entrepreneurship **i.e.** engage in innovative activities by using creative thinking to envision better ways of accomplishing professional goals, thus potentially preparing students for the unknown and responding to changes in scopes of practice, different models of practice and responding to unstructured complex problems. This may indicate a potential tension between pharmacy’s professional aspirations and the educational foundation such that these aspirations may be met. 

Given the increasing emphasis on inter-professional teamwork in healthcare [29], it is pertinent to note that the GbCF doesn’t reference teamwork. Similarly, leadership is not referenced in the GbCF in spite of broad acknowledgment across health systems that health practitioners need skills and abilities to lead complex change [30]. Both teamwork and leadership are referenced in learning outcome statements from three of the four jurisdictions examined in this paper. 

Agreement in most outcomes and the GbCF reinforces the view of pharmacists as patient focussed medicines experts across jurisdictions examined. Each jurisdiction has a different articulation, but the precepts of the learning outcome are essentially the same. It is also important to note that a learning outcome statement may be based on the same principles, for example, “Clinically evaluate the appropriateness of prescribed medicines” but the level of achievement of the required knowledge, skills and attributes could vary dramatically dependent on patient and medication complexity.

These comparisons and contrasts provide insights for consideration in future revisions of individual jurisdiction’s outcomes documents and consideration regarding transferability and harmonization of qualifications, and hence mobility, across these jurisdictions. These comparisons also provide impetus for cross-border institutional networking in a thoughtful and purposeful manner potentially to generate a dependable set of assessment tools across national borders developing a common metric for outcome assessment irrespective of different program delivery. No published work addressing a common assessment tool in pharmacy education could be located; however, given the data presented in this paper development of common assessment tools is clearly feasible.

In the United States, the National Association of Boards of Pharmacy has developed the Pharmacy Curriculum Outcomes Assessment (PCOA), a validated evaluation tool available for schools of pharmacy. Subject matter experts and pharmacy faculty members from across the United States develop PCOA questions [31] designed to evaluate foundational knowledge and whether or not a school’s curriculum has adequately addressed all desired curricular content, as reflected by the performance of students collectively on the exam; however, there is no published reference to this test being used internationally. The use of the United States Foundations of Medicine Clinical Science Examination has recently been reported in a collaboration across five Australian medical schools [32]. Other health professional disciplines have developed competency based tools for outcome assessment in more than one jurisdiction. For example, in speech pathology the COMPASS^®^ tool [33] is used in speech pathology programs in Australia, New Zealand, Hong Kong and Singapore, in spite of differences in competencies in these jurisdictions. 

The comparisons of expected outcomes displayed in this paper clearly demonstrate the expectation that pharmacy curricula in Australia, Canada, the United Kingdom and the United States align with pharmacists as patient-orientated medicines experts. This is apparent irrespective of degree title. What is also evident from this analysis is the close relationship between the professional and university systems, as reflected by competencies and learning outcomes respectively, in these four jurisdictions.

## Figures and Tables

**Table 1:** Content analysis of high-level outcomes from Australia, Canada, the United Kingdom and United States and GbCF.

FIP Global Framework [24]	Australia [16]	Canada [17,18]	United Kingdom [11]	United States [23]
**1. Pharmaceutical Public Health Competencies** 1.1. Health promotion 1.2. Medicines information and advice	Apply pharmaceutical, medication and health knowledge and skills Within their scope of practice, in the assessment of individual health status and medication needs, and where necessary, develop, implement and monitor management plans in consultation with patients/clients and other health professionals to improve patient outcomes. To promote and optimise the health and welfare of communities and/or populations.	As **Care Providers** pharmacy graduates use their knowledge, skills and professional judgement to provide pharmaceutical care and to facilitate management of patient’s medication and overall health needs. As **Advocates** pharmacy graduates use their expertise and influence to advance the health and well-being of individual patients, communities, and populations, and to support pharmacist’s professional roles. As **Scholars** pharmacy graduates have and can apply the core knowledge and skills required to be a medication therapy expert, and are able to master, generate, interpret and disseminate pharmaceutical and pharmacy practice knowledge.	Validating therapeutic approaches and supplying prescribed and over-the-counter medicines. Identify and employ the appropriate diagnostic or physiological testing techniques in order to promote health.	Health and wellness (**Promoter**)—Design prevention, intervention, and educational strategies for individuals and communities to manage chronic disease and improve health and wellness. Educator (**Educator**)—Educate all audiences by determining the most effective and enduring ways to impart information and assess understanding. Population-based care (**Provider**)—Describe how *population-based care* influences *patient centred care* and influences the development of practice guidelines and evidence-based best practices. Learner (**Learner**)—Develop, integrate, and apply knowledge from the foundational sciences (i.e., *pharmaceutical*, *social/behavioural/administrative*, and *clinical sciences*) to evaluate the scientific literature, explain drug action, solve therapeutic problems, and advance population health and *patient centred care*. Patient Advocacy (**Advocate**)—Assure that patients’ best interests are represented. Cultural sensitivity (**Includer**)—Recognize *social determinants of health* to diminish disparities and inequities in access to quality care.
**2. Pharmaceutical Care Competencies** 2.1. Assessment of medicines 2.2. Compounding medicines 2.3. Dispensing 2.4. Medicines 2.5. Monitor medicines therapy 2.6. Patient consultation and diagnosis	Apply pharmaceutical, medication and health knowledge and skills. Within their scope of practice, in the assessment of individual health status and medication needs, and where necessary, develop, implement and monitor management plans in consultation with patients/clients and other health professionals to improve patient outcomes. To promote and optimise the health and welfare of communities and/or populations. Formulate, prepare and also supply medications and therapeutic products.	Under Role of **Scholar:** Pharmacy graduates have and can apply the core knowledge and skills required to be a medication therapy expert, and are able to master, generate, interpret and disseminate pharmaceutical and pharmacy practice knowledge. As **Care Providers** pharmacy graduates use their knowledge, skills and professional judgement to provide pharmaceutical care and to facilitate management of patient’s medication and overall health needs. Under role of **Care provider:** develop a care plan that addresses a patient’s medication-therapy problems and priority health and wellness needs. dispense a medication according to a new prescription; dispense an authorized refill of a medication. As **Advocates** pharmacy graduates use their expertise and influence to advance the health and well-being of individual patients, communities, and populations, and to support pharmacist’s professional roles.	Validating therapeutic approaches and supplying prescribed and over-the-counter medicines. Analyse prescriptions for validity and clarity. Clinically evaluate the appropriateness of prescribed medicines. Working with patients and the public. Establish and maintain patient relationships while identifying patients’ desired health outcomes and priorities.	Patient-centred care (**Caregiver**)—Provide *patient-centred care* as the medication expert (collect and interpret evidence, prioritize, formulate assessments and recommendations, implement, monitor and adjust plans, and document activities). Health and wellness (**Promoter**)—Design prevention, intervention, and educational strategies for individuals and communities to manage chronic disease and improve health and wellness. Population-based care (**Provider**)—Describe how *population-based care* influences *patient centred care* and influences the development of practice guidelines and evidence-based best practices. Learner (**Learner**)—Develop, integrate, and apply knowledge from the foundational sciences (i.e., *pharmaceutical*, *social/behavioural/administrative*, and *clinical sciences*) to evaluate the scientific literature, explain drug action, solve therapeutic problems, and advance population health and *patient centred care*. Patient Advocacy (**Advocate**)—Assure that patients’ best interests are represented.
**3. Organisation and Management Competencies** 3.1. Budget and reimbursement 3.2. Human Resources management 3.3. Improvement of service 3.4. Procurement 3.5. Supply chain and management 3.6. Work Place Management		As **Managers** pharmacy graduates use management skills in their daily practice to optimize the care of patients, to ensure the safe and effective distribution of medications, and to make efficient use of health resources.	Ensuring that safe and effective systems are in place to manage the risk inherent in the practice of pharmacy and the delivery of pharmaceutical services. Manage and maintain quality management systems including maintaining appropriate records. Procure and store medicines and other pharmaceutical products working within a quality assurance framework.	Medication use systems management (**Manager**)—Manage patient healthcare needs using human, financial, technological, and physical resources to optimize the safety and efficacy of medication use systems.
**4. Professional/Personal Competencies** 4.1. Communication skills 4.2. Continuing Professional Development (CPD) 4.3. Legal and regulatory practice 4.4. Professional and ethical practice 4.5. Quality Assurance and Research in the work place 4.6. Self-Management	Communicate in lay and professional language, choosing strategies appropriate for the context and diverse audiences. Demonstrate professional behaviour and accountability in the commitment to care for and about people. Retrieve, critically evaluate and apply evidence in professional practice. Reflect on current skills, knowledge, attitudes and practice; planning and implementing for ongoing personal and professional development. Make, act on and take social responsibility for clinically, ethically and scientifically sound decisions	As **Communicators** pharmacy graduates communicate with diverse audiences, using a variety of strategies that take into account the situation, intended outcomes of the communication and the target audience. As **Professionals** pharmacy graduates honour their roles as self-regulated professionals through both individual patient care and fulfilment of their professional obligations to the profession, the community and society at large. Under role of **Professional:** Maintain their competence to practice through lifelong learning. Under role of **Professional:** Practice in an ethical manner which assures primary accountability to the patient.	Expectations of a pharmacy professional. Communicate with patients about their prescribed treatment. Recognise ethical dilemmas and respond in accordance with relevant codes of conduct. Recognise personal health needs, consult and follow the advice of a suitably qualified professional, and protect patients or the public from any risk posed by personal health. Maintaining and improving professional performance Review and reflect on evidence to monitor performance and revise professional development plan. Reflect on personal and professional approaches to practice.	Communication (**Communicator**)—Effectively communicate verbally and nonverbally when interacting with an individual, group, or organization. Problem Solving (**Problem Solver**)—Identify problems; explore and prioritize potential strategies; and design, implement, and evaluate a viable solution. Self-awareness (**Self-aware**)—Examine and reflect on personal knowledge, skills, abilities, beliefs, biases, motivation, and emotions that could enhance or limit personal and professional growth. Professionalism (**Professional**)—Exhibit behaviours and values that are consistent with the trust given to the profession by patients, other healthcare providers, and society.
**No comparable competency in the FIP Global Framework**	Demonstrate team and leadership skills to deliver safe and effective practice	As **Collaborators** pharmacy graduates work collaboratively with teams to provide effective, quality health care and to fulfil their professional obligations to the community and society at large.		Interprofessional collaboration (**Collaborator**)—Actively participate and engage as a healthcare team member by demonstrating mutual respect, understanding, and values to meet patient care needs. Leadership (**Leader**)—Demonstrate responsibility for creating and achieving shared goals, regardless of position.
				Innovation and Entrepreneurship (**Innovator**)—Engage in innovative activities by using creative thinking to envision better ways of accomplishing professional goals.

Note: Text in Table 1 is taken directly from references [11,16,17,18,23,24].
